# Preparing for the next pandemic: Reflections and recommendations from Florida

**DOI:** 10.1371/journal.pone.0314570

**Published:** 2024-12-02

**Authors:** Anicca Liu, Rachel N. Waldman, Bonnie Deal, Johnathan Duff, Jacob N. Batycki, Ernesto A. Pretto, Jorge Saavedra, José Szapocznik

**Affiliations:** 1 Department of Public Health Sciences, University of Miami Miller School of Medicine, Miami, Florida, United States of America; 2 School of Social Policy & Practice, University of Pennsylvania, Philadelphia, Pennsylvania, United States of America; 3 Department of Communication Studies, University of Miami, Coral Gables, Florida, United States of America; 4 Department of Anesthesiology, University of Miami Miller School of Medicine, Miami, Florida, United States of America; 5 AHF Global Public Health Institute, Fort Lauderdale, Florida, United States of America; CAMS PUMS IMI: Chinese Academy of Medical Sciences & Peking Union Medical College Institute of Medical Information, CHINA

## Abstract

**Context:**

The COVID-19 emergency warrants reflection on how to improve future infectious disease pandemic preparedness and response (PPR). U.S. States took diverse approaches to COVID-19, with Florida’s approach characterized by fewer restrictions on businesses and individuals. Despite the profound effects of the pandemic, there is a dearth of stakeholder-informed recommendations for PPR at the state level. This study aims to empirically examine stakeholder perspectives on PPR in Florida.

**Methods:**

25 semi-structured interviews were conducted with former and current leaders from government, academia, and the private sector in Florida. Participants were asked about challenges encountered during COVID-19 and considerations for what should be done for future pandemics. Interview transcripts and notes were analyzed using qualitative content analysis (QCA).

**Findings:**

Results were organized into four main categories (*recommendations* for future actions, *challenges* in PPR, *successes* and *failures* during the COVID-19 response), across which six sub-categories were identified: public health systems capacities; mitigation of disease transmission; roles and relationships; messaging and information dissemination; minimizing other adverse effects of a pandemic; and public health culture. Considering the neglect of existing pandemic plans and jurisdictional tensions around decision-making during COVID-19, participants proposed implementing a pandemic playbook that delineates the responsibilities of relevant agencies and processes of waiving standard procedures. While many suggested closures and restrictions to avoid the spread of disease, others questioned the extent to which such strategies should be implemented.

**Conclusions:**

This study corresponds with the need for consensus-building across ideological divisions, revealing tensions among federal, state, and county-level entities, as well as across state-level agencies. Participants defined successful pandemic response as not only comprising the mitigation of disease transmission, but also the minimization of adverse social and economic effects. Participants discussed strategies for a unified, well-coordinated approach to future pandemics that balances health and economic concerns.

## Introduction

The COVID-19 emergency demonstrates the need for strategic reflection on how to better prepare for and respond to future infectious disease pandemics. Within a federalist governance structure, individual U.S. states have the primary responsibility for public health activities, including responding to emergencies [1–4]. States took diverse approaches to the COVID-19 crisis [5–7]. For example, some states, such as Nevada and Hawaii, used a cooperative state-local approach, which empowered local (i.e., county and municipal) governments to exercise greater autonomy [8, 9]. Others pursued an emergency response centralized around governors’ executive orders and leadership [8, 9] The State of Florida initially took a decentralized approach, in which local governments were permitted to propose policies to respond to COVID-19 [9, 10]. Over time, however, local decision-making was restricted [5, 9]. In May 2021, Florida Governor Ron DeSantis suspended local COVID-19 policies and prohibited new mandates from being enacted on the local level, situating decision-making at the state executive level [11]. Evaluations of the U.S. response to COVID-19 have found that states with greater preemption of local authorities, such as Florida [12], implemented fewer COVID-19 policies at the local and state level, whereas states with less preemption generally “demonstrated a more comprehensive response to the pandemic” [13].

U.S. states also differed in their approaches to ease initial COVID-related restrictive policies [14, 15]. For example, some states factored in vaccination rates when determining when to roll back restrictions such as stay-at-home orders and closures, while others, such as Florida, began re-opening the state prior to the availability of vaccines [6]. Since Florida’s economy depends greatly on its hospitality industry [16, 17], decision-makers weighed the state’s economic wellbeing along with public health considerations in determining the state’s COVID-19 response [5, 17]. Relative to other states, Florida’s approach was characterized by fewer restrictions on businesses and individuals [5]. For example, Florida avoided enacting a statewide mask mandate throughout the pandemic [14] and began the first phase of reopening the state in May 2020—earlier than many other states [18, 19]. Research suggests that state-level responses to the COVID-19 pandemic, particularly those surrounding the implementation of non-pharmaceutical interventions (NPIs), were partially driven by political affiliation [20–22]. On average, as compared to Democratic governors, Republican governors, with some exceptions, implemented social-distancing guidelines later [1, 6, 23], enacted less restrictive policies [20] and were less likely to keep public health measures in place after COVID-19 vaccines became widely available [6, 22, 24].

The different approaches state governments took to addressing the pandemic may have implications for outcomes relevant to morbidity and mortality. Emerging evidence suggests that states that implemented more disease mitigation measures, such as containment and closure policies and mask mandates, had fewer COVID-19 cases, hospitalizations, and deaths [25–30]. While COVID-19 attributed deaths were initially higher among states with Democratic governors during the first few months of the pandemic when policies were mostly uniform across states, the trend quickly reversed as states with Republican governors began reporting higher death rates, per capita cases, and positive tests [31–33]. This was attributed, in part, to the growing political polarized response, which prompted the early relaxation of mitigation measures among some states [33]. Despite concerns around the potential trade-offs of health and economy posed by policy mandates, Bollyky et al. found that no COVID-19 policies were associated with changes in state-level GDP [34, 35]. Nonetheless, restaurant closures and mask use, tied with reduced COVID-19 cases and deaths, were associated with a lower proportion of employment [34, 35].

It is necessary to build upon lessons learned from Florida’s specific policy context to improve pandemic preparedness and response (PPR) at the state level. While recommendations for future pandemic preparedness have been established by various commissions on the national and global level [36–40], few states have pursued stakeholder-guided processes for longer term PPR planning, instead focusing on establishing advisory bodies to guide acute responses to the COVID-19 emergency. In particular, the State of Florida has pursued some education and business-focused reflection through the governor’s *Re-Open Florida Task Force* [19]. However, this group, which was established to present plans for re-opening Florida’s businesses and public spaces, was comprised mostly of business executives and lacked public health perspectives [19].

There remains a dearth of stakeholder-informed recommendations specific to U.S. states, especially around decisions and actions to prepare for and respond to future pandemics at the state level. In particular, empirical work garnering stakeholder perspectives on challenges from COVID-19 and recommendations for future state PPR has been limited [41–43]. To date, no studies have represented the views of stakeholders in recommendations for enhancing pandemic preparedness and response in Florida. To address the gap in stakeholder-informed research on PPR in Florida, this qualitative study aims to distill lessons learned from Florida’s COVID-19 response to inform future pandemic policies and practice. Therefore, we gathered perspectives on challenges and recommended actions for future pandemic PPR from stakeholders involved in the Florida’s pandemic response, including officials from government, the private sector, and academia. Drawing from stakeholder perspectives and experiences during the pandemic, this study also serves to provide an assessment of the state’s COVID-19 response.

## Methods

### Study design and participants

**Fig 1:**
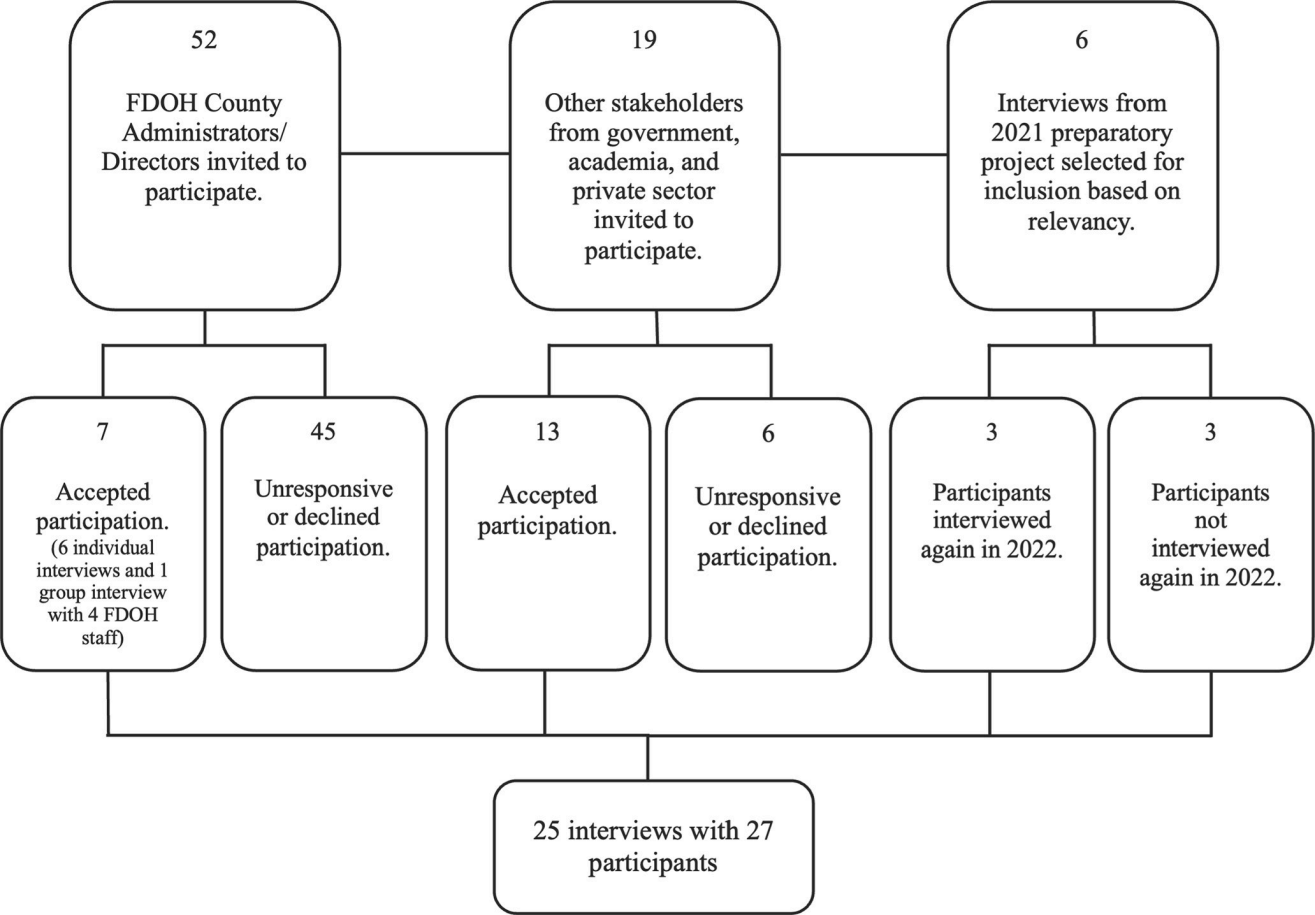
Participant recruitment.

This study used qualitative methods to gather perspectives on pandemic preparedness and response from stakeholders in government, academia, and the private sector in Florida (see Table 1). Participants were recruited from January 25, 2021 to December 7, 2022. Initially, purposive sampling was used to invite all 52 Florida Department of Health (FDOH) County Directors and Administrators (representing the 67 Florida counties) to participate in interviews. Seven accepted, resulting in 7 interviews, including one group interview with 4 additional FDOH staff, equating to an approximate 13.46% acceptance rate from our intended sample. Due to challenges in obtaining interviews with county FDOH Directors/Administrators, we expanded our sample to include greater diversity of perspectives from different sectors. We took advantage of initial set of participants’ recommendations to include perspectives from stakeholders who had a significant role in responding to COVID-19 or prior emergencies, including specific individuals to interview. This resulted in a participant sample comprising of public health officials, public health experts in academia and the private sector, as well as legislators and industry leaders with an interest or experience in public health (see Fig 1).

**Table 1:** Participant affiliation.

Sector	Gender		Total
	M	F	
**Government**	**10**	**9**	**19**
Florida Department of Health (state and county level)	5	8	
Florida Division of Emergency Management/FEMA	2	0	
Members of the Florida Legislature	3	1	
**Academia**	**1**	**2**	**3**
**Private Sector**	**3**	**2**	**5**
Disaster management	1	1	
Hospitality	2	1	
**Total**	**14**	**13**	**27**

### Interviews

Semi-structured interviews were used to collect input from participants on PPR in Florida. Participants were asked questions about challenges faced during the COVID-19 pandemic, and considerations for what the state of Florida should do the same or differently for future pandemics (see S1 File). Potential participants were invited to interview through letters sent via email that explained the purpose of the study, asked for confirmation of their participation, and informed them that confidentiality would be maintained and their names would not be connected to their responses. All participants responded to the invitations via email, agreeing to be interviewed and participate in the study. In addition, at the beginning of the interview session, participants were reminded of the confidentiality of their responses, and they provided consent to being recorded prior to the formal interview. Interviews were conducted on a video conferencing platform and were between 20–65 minutes in length. Nineteen interviews were conducted with 24 participants (2 group interviews and 17 individual interviews) between February and December 2022. Six previously recorded interviews from another component of the larger study on lessons learned from COVID-19 were also included in this study for a total of 25 interviews with 27 participants included for analysis (6 interviews from the preparatory project in 2021 and 19 from 2022). Of the 25 interviews, 22 were recorded and transcribed by a professional and HIPAA compliant transcription service; detailed notes were taken for the remaining 3 interviews. All data were stored in a secure server and were anonymized through the provision of participant IDs to conceal identities. The University of Miami Institutional Review Board (IRB) determined that the proposed activity is not research involving human subjects as defined by DHHS and FDA regulations, and therefore, that IRB review and approval was not required.

### Analysis

Interview transcripts and notes were analyzed using qualitative content analysis (QCA) [44, 45] using NVivo qualitative data analysis software [46]. Content analysis is a method to classify written or oral materials into identified categories of similar meanings [45]. Rather than focus on building theory or finding relationships among categories, QCA focuses on extracting categories from the data—here the interpretation of participants’ responses regarding PPR [47, 48]. The process of data analysis using QCA followed a two-phase procedure: a pilot phase to create the coding framework and the main analysis phase which applies this iterative framework to remaining data. The steps in data analysis included: (a) defining the units of coding, (b) open coding of the data, (c) identifying the main categories, (d) selective coding to establish the coding frame, (e) defining subcategories and coding rules, and (f) coding the remaining data [44].

In this study, individual interview transcripts represented coding units. The first phase of coding involved open coding. Open coding involves reviewing the data and identifying any concepts that appear relevant [47]. Data units—such as participants’ comments—are marked with an initial code capturing their meaning or interpretation. Coding can be an interpretation from the researcher, or *in-vivo*, meaning using the respondents’ own language. Four representative transcripts were selected and coded independently by four team members (AL, RW, BD, JD). Team members then convened to review initial coding and determine which concepts made suitable main categories. Through these discussions, four main categories emerged: *recommendations*, *challenges*, *successes*, and *failures*. The remaining 21 transcripts and notes were coded independently by two team members each. Relevant comments were coded into one of the four main categories and a summary interpretation of the data unit was determined. The two coders for each transcript met consistently throughout the process to verify coding alignment, eliminate duplication, and synthesize the summary interpretation of quotes.

Once all relevant data was coded by main category, the four coders developed subcategories in the coding frame. Initial subcategories were developed using data from the *recommendations* main category as it had the most data and was most pertinent to the research question. Once all subcategories were established and defined in the coding frame, data from the remaining three main categories was analyzed. Modification of the coding frame and subsequent data analysis remained a flexible, iterative process. When differences in analysis occurred, the two coders of that transcript discussed it with the other two coders until a consensus was reached. Double coding was permitted when data units possessed multiple meanings or were relevant to more than one category or subcategory.

## Results

Participant interviews focused primarily on four categories: *recommendations* for PPR, *challenges* in PPR, and examples of *successes* and *failures* during the COVID-19 pandemic response. *Recommendations—*followed closely by *challenges*—had the highest frequency of codes. This reflected participants’ detailed considerations on future actions for improving the state’s response to emerging infectious disease threats and perceived difficulties faced in PPR. Notably, participants discussed considerations for PPR beyond those in the purview of state government authority, including global, national, and county-level actions.

Further analysis revealed that the four categories (*recommendations*, *challenges*, *successes*, and *failures*) spanned across six subcategories, outlined in Table 2. In this manuscript, we introduce a broad framework of the priority areas for PPR, focusing primarily on *challenges* and *recommendations*, with selected examples of *successes* and *failures* provided where relevant. Table 2 presents a description of the priority areas (as subcategories) and corresponding topics (including those that are not fully explored in this paper). A thorough exploration of all subcategories, and detailed examples of successes and failures are beyond the scope of this paper.

**Table 2:** Description of subcategories.

Sub-Category	Definition	Topics
**Public Health Systems Capacities**	The capacities needed to ensure strong, resilient, responsive, and well-resourced public health systems adequately prepared for future pandemics	Public health funding
		Workforce, training, and professional development
		Testing and laboratory capacities
		Production of materials and access to resources
		A guiding pandemic “playbook”
		Adequate technologies
**Mitigation of Disease Transmission**	The policies and strategies needed to mitigate the transmission or severity of communicable disease and the context in which they should be implemented	Travel restrictions and closures
		Implementation of public health emergency policies
		Distribution of tests, vaccines, and treatments
**Roles and Relationships**	The responsibilities and authorities of specified actors, including federal, state, and county-level government before and during a pandemic, and a network of partnerships that includes all relevant stakeholders	Responsibilities and authorities of federal, state, and county-level governments
		Relationships and multisectoral representation
**Messaging and Information Dissemination**	The framing and dissemination of information during a pandemic, including strategies to ensure the public is receptive to the message and the messenger	Framing of messages
		Dissemination of data and recommendations
		Trustworthy and autonomous messenger(s)
**Minimizing Other Adverse Effects of a Pandemic**	Minimizing the social and indirect health consequences that may result from a pandemic or an associated intervention	Economic effects of the pandemic or of mitigation efforts
		Other health considerations not associated with the pathogen
**A Culture of Public Health**	The cultivation of a culture that prioritizes public health and encourages trust in science to promote effective implementation of and compliance with public health guidelines	Prioritization of public health
		Trust in science, public health, and government

### Public health systems capacities

#### Funding

Similar to public health professionals throughout the country, participants from Florida Department of Health (FDOH) and Florida Division of Emergency Management (FDEM) spoke to the importance of sustained financial investment in the public health system. Adequate readiness, they said, depends on public health and medical systems that are well-staffed and properly funded. Low funding was identified as a factor that weakened Florida’s public health systems and inhibited the state’s ability to effectively respond to COVID-19:

“Frankly we have cut our health departments to the bone. And they’re now on the front lines of responding to a pandemic, setting up testing and now setting up vaccines without providing them any more resources… I would say from a public health perspective, it’s been a strain.” (member of Florida Legislature)

According to one county FDOH Director, public health budgets had been historically “gutted” at the state level, resulting in challenges to building the public health capacities needed for an effective response. This participant, among others in FDOH, emphasized the importance of sustained investment in county health departments. Interviews elucidated that during the early response to COVID-19, the lack of certainty around states’ ability to carry out pandemic responses due to budgetary constraints led to insufficient responses at the state-level. One participant, a FDEM leader, noted that during COVID-19, the federal government did not immediately declare full reimbursement for state pandemic spending, causing some states to delay their response until adequate funding could be secured [49]:

“Money dictates resources and if the states and local governments are worried about who is going to pay for it …They wait to see how much the federal government is going to pay before they start buying things, especially in states that don’t have a lot of money.” (FDEM leader)

Responding to this challenge, that participant recommended that the federal government immediately ensure full reimbursement at the onset of future pandemics.

#### Workforce and resources

Low funding in public health was identified by several participants from FDOH and FDEM as a catalyst for other barriers, including the lack of an adequate workforce. Speaking to staffing shortages at county health departments during the pandemic one county FDOH Director explained [50]:

“As we started getting cases and we started doing our surveillance, we very quickly realized that we were going to be overwhelmed because public health has never been more short-staffed…just really really cut significantly to the very bare minimum.”

Beyond experiencing a shortage of personnel, four participants from FDOH noted the lack of training for basic pandemic mitigation activities, such as contact tracing and administration of diagnostic tests and vaccinations. Several of these participants shared specific ideas on how to build a well-prepared workforce, such as establishing a statewide workforce training curriculum ahead of the next pandemic. To address shortages, some mentioned partnering with universities to engage medical students in public health interventions, as was the case in one county where the FDOH deputized medical students and residents to help with contact tracing.

Inadequate funding, combined with the limited national production of public health goods, was noted to be linked to issues surrounding the procurement and stockpiling of resources [51]. One participant from FDEM stated, “[a] lesson learned is the cupboard cannot be bare going into every [pandemic].” Stakeholders from both public health and emergency management suggested that the lack of public health goods ignited competition for resources between states during COVID-19, inhibiting a timely and adequate response. Therefore, they supported increasing U.S. production of public health goods and establishing pre-negotiated platforms for state resource needs.

#### Testing and laboratory capacities

In addition to personnel required, several participants from FDOH and FDEM discussed testing and laboratory capacities. Nearly all participants deemed the lack of adequate and timely testing at the onset of COVID-19 as a major shortcoming of the national and county-level responses. Three participants from FDOH spoke about being notified that their Epidemiology and Laboratory Capacity funding would be jeopardized if they did not use tests approved by the CDC. They believed this restriction, coupled with the recall of early CDC-approved tests, caused a month-long gap in surveillance capacity:

“We were told that if we ran our own test that this would jeopardize our ELC funding…And so we sat on our hands literally from February 2^nd^ until February 28^th^ when the CDC approved the test. So that’s a gap of almost a month where we could not track the virus because we were not allowed to do testing. The WHO released their test kit at the end of January. So, countries all around the world were doing testing except for the United States.” (state-level FDOH leader)

To establish adequate capacities ahead of a future pandemic, a few county FDOH Directors suggested granting greater flexibility to academic institutions and state laboratories for diagnostic test development during emergencies. Also, it was recommended that pre-planned capacity for state laboratories be established ahead of a future pandemic.

#### A pandemic “playbook”

Participants from FDOH and FDEM highlighted the need for public health and emergency response systems to collaboratively improve and execute a unified “playbook” across the state for public health emergencies. One FDEM leader noted that a Florida state plan existed but was abandoned during COVID-19, stating, “one of the first things that the State of Florida did, and other states did, is they immediately went away from the pandemic plan.” Public health and emergency management stakeholders stressed that a playbook that effectively outlines the state’s PPR plan should be adhered to in the event of a future public health emergency. In particular, participants from emergency management, public health, the state legislature, and academia suggested that the playbook may include strategies for determining vulnerable populations to prioritize for public health interventions and identifying brick-and-mortar sites for test and vaccine administration. Referencing Florida’s established ability to quickly limit bureaucratic processes when responding to hurricanes, emergency management participants from government and the private sector suggested preemptively identifying laws, statutes, and limitations that should be waived in the event of a public health emergency, to facilitate a timely response.

### Mitigation of disease transmission

#### Restrictions and closures

Participants across all sectors spoke about specific strategies to mitigate the transmission and severity of a communicable disease during an outbreak. Discussion mostly centered on pandemic policies that restrict movement, behavior, and maximum occupancy in public and private spaces. With consideration to the state’s limited implementation of closures and containment measures, several participants, including those from state legislature, health departments, emergency management, and academia, recommended mask mandates and social distancing measures in the event of a future airborne threat. Additionally, some from the state legislature, academia, and disaster management stressed the need for closures or restrictions on maximum spatial capacity in “hot spots” for virus transmission, such as bars and restaurants, especially before a vaccine becomes widely available to the public:

“It’s limitations on specific activities and industries and businesses that we know are going to be hotbeds for transmissions…there’s no reason that we cannot have reasonable restrictions on bars and nightclubs. That doesn’t mean we have to shut them down. It means there should be strict masking requirements, strict requirements on social distancing…on capacities of bars…We can’t just let them get packed like this, the way that they have allowed in the state of Florida…” (member of Florida Legislature)

Florida’s abundant tourism industry and porous borders also sparked suggestions around travel restrictions and policies that would promote containment at entry points to the state.

#### Implementation of policies

The topic of mitigation strategies prompted varied responses on the timing of restrictive measures and the extent to which they should be implemented. Some participants from emergency management, public health, and the state legislature, for example, pointed to Florida’s lack of a mask mandate and early relaxing of restrictions on businesses as evidence that “not enough was done.” These participants advocated for a quick response, including the implementation of early mitigation actions based on information from early alerts in other countries.

On the other hand, some participants from the state legislature and hospitality industry described certain responses to COVID-19 as excessive. They were joined by stakeholders across public health and emergency management on the need for more context-specific and flexible interventions. According to a few leaders from public health and the hospitality industry, the response should include an initial deference to caution through the implementation of stringent policies, followed promptly by a relaxation of restrictions when better knowledge on the virus emerges. One county FDOH Director stated, “start off aggressive and then work your way down instead of working your way up.” Some also argued that in implementing lockdowns, and when considering vaccine strategies, interventions should target vulnerable groups rather than the entire population. Participants across all sectors we included in this study believed these flexibilities would balance science and economic interests and would be more palatable to the public. As one public health academic stated:

“What’s required is a careful balance…If you are in the midst of a surging epidemic, then yes, you do need to put in place certain interventions. But I think all of us have become acutely aware of the fact that there is a public level of tolerance for certain interventions which is greater than others.”

### Roles and relationships

#### Roles and authorities

Almost every participant emphasized the importance of a coordinated and collaborative effort among all stakeholders and across government levels, agencies, and partners. In reflecting on their experiences during COVID-19, leaders from public health and emergency management recalled tensions between federal, state, and local governments that inhibited an effective pandemic response. Several participants from FDOH expressed concerns surrounding the limited decision-making capabilities of local governments as compared to the state. Regarding the state’s exclusion of local input, one hospitality industry leader noted:

“In this instance, a governor, if they feel like they don’t need the counties and they’re going to press forward with their agenda with or without the counties on board, that is not a helpful posture. Do they have the political capital and/or political horsepower to do that? In all likelihood, yes. But is it effective? Probably not.”

Participants from FDOH and FDEM spoke about disagreements between the state and local governments, resulting in the Florida governor’s removal of local powers to implement COVID-19 restrictions. Interviews highlighted that local FDOHs were also limited in their autonomy as members of a centralized health system that was required to follow guidance from the state.

In addition, leaders in public health and emergency management mentioned that there were “turf” conflicts among state-level agencies, specifically the FDOH and the FDEM, resulting in a weak supply chain and delays in testing:

“Personally, I think there was not good communication between DOH and DEM. And that hurt things or slowed things down…We had to rely on our state emergency management. It put a delay in things as far as getting out there, getting the testing. (county FDOH Director)

According to participants from FDOH and FDEM, these conflicts likely stemmed from poor coordination between agencies as well as disagreement over which agency should take lead in a pandemic:

“Frankly the governor put a lot of this management over to Department of Emergency Management. DEM led this at the state level rather than DOH. So, to me it felt more like a military operation than a public health operation and I think you need both.” (county FDOH Director)

“A lot of public health officials have no earthly idea [about] the role of emergency management, because they treat it like a disease outbreak and not as a national or state or localized emergency…” (FDEM leader)

To alleviate these tensions, several leaders from public health and emergency management recommended the establishment of a unified state-level collaborative framework that explicitly integrates FDOH and FDEM responses.

While participants had diverse opinions on who should lead the public health response, nearly all emphasized the need for each actor to clearly understand their responsibilities within the larger network. Some participants, namely directors of county health departments, stressed the need to ensure greater decision-making power for FDOH and local leaders, considering their exceptional knowledge of their respective communities. Others from the hospitality industry and the state legislature, however, favored the centralization of state authority.

#### Relationships and representation

Drawing from positive experiences during COVID-19, several county FDOH Directors discussed collaborative relationships and community representation as essential to creating and maintaining an effective pandemic response. These participants emphasized the importance of building relationships with community leaders and stakeholders, such as universities, health councils and local elected officials, before a public health emergency emerges. One county FDOH Director relayed that “creating those relationships and that trust absent the duress of a crisis is critical to establishing effective relationships to deal with the crisis.” Many credited these pre-established partnerships with providing needed support to county FDOHs for testing and contact tracing. Alternatively, when communities had no prior relationship with county health agency leaders, trust and coordination was negatively impacted.

Participants across all sectors included in this study stressed the need to bring all relevant stakeholders in a community “to the table” to ensure adequate representation and to develop a multisectoral and integrated approach to the pandemic response. For instance, one county FDOH Director shared that “it truly is a community-wide effort because it’s impossible for one organization in this community to be responsible for meeting all the needs that we have.” In particular, participants stressed that leaders from think tanks and industries such as banking, travel, and logistics, be adequately represented in PPR.

### Messaging and information dissemination

#### Framing

Nearly all participants described major challenges associated with communication during the COVID-19 pandemic, identifying barriers to the public’s reception of both the message and the messenger. Regarding the framing of the messages disseminated to the public, several participants from FDOH, the hospitality industry, and the state legislature shared concerns around the lack of cohesive communication across government sectors. These concerns were grounded in contrasting perspectives on the severity of COVID-19, the elevation of “too many voices” circulating incongruent messages to the public, and the constantly evolving information associated with the unknowns of a novel pathogen:

“[In Florida, there were] too many people making decisions and getting in front of the cameras, and having press conferences, and it’s just confusing. One mayor and one city, and then the city two blocks away [has] a whole different approach. And it’s not [a] cohesive plan at all.” (member of Florida Legislature)

A few participants from the private sector (disaster management and hospitality industry) and state legislature also commented on the negativity bias in public communication such as emphasis on risks and often-used inflammatory tone. These participants believed this prompted hysteria among some, and fatigue among others. One member of the Florida legislature remarked, “I think once people are so tired of this right now, I’m afraid…everybody’s just going to go back to worse than where we were before.” In the event of a future pandemic, several public health participants from FDOH and academia emphasized the need for bipartisan agreement on a unified message conveyed in a positive fashion. To be well-received by the public, messages need to be communicated consistently across the state and inspire hope.

#### Information and dissemination

Stakeholders from public health supported the need for high-quality, real-time information available to the public. According to some participants from academia and FDOH at the state-level, transparency in messaging was complicated during COVID-19 by purposeful misinformation based on a “highly flawed analysis” of data. The FDOH was referred to as having released an advisory that opposed the COVID-19 vaccination of healthy children, contradicting the medical advice of most public health institutions [52]. One participant from FDOH at the state-level remarked “we’re the only state in the country [that] advises against giving children COVID-19 vaccines…It goes against all of the medical advice at every institution and every level of government…” To combat misinformation, participants from public health, academia, government, and the hospitality industry proposed that public officials emphasize honesty about the knowns and unknowns of the virus, and to be transparent with the public when decisions are made “out of an abundance of caution.”

Additionally, participants from FDOH at the state and local levels highlighted the lack of consistent information available after the state COVID-19 public data dashboard was removed during the pandemic [53]. These participants identified this early COVID-19 information dashboard as an example of an initial success in this area. After the dashboard was taken down, however, they expressed concerns that the public lacked a data source they had previously relied on to make informed decisions:

“We started off very transparent when we had data in the dashboard… helping people frame day to day what was going on with the pandemic. And they got very attached to that and a lot of people were making decisions based off of that and when that [was removed], I felt like it was harmful to the community…I think people just felt like they had a better sense of ownership, they could make decisions…for that [not to be] sustained throughout the response. I think that was harmful.” (county FDOH Director)

Therefore, a forum that addresses public needs, provides real-time data, and is consistently available was suggested to foster transparency and ensure the public has access to reliable, reputable information throughout a public health emergency.

#### An honest and trustworthy messenger

Transparency was noted as an important trait for the messengers communicating with the public during a pandemic. Despite complaints surrounding an abundance of voices, several public health participants identified COVID-19 messaging as “tightly controlled” by state government leaders, which they believed caused county FDOHs, the former Florida Surgeon General, and public universities to be limited in their speech to the public. One county FDOH Director noted:

“Messaging was very tightly controlled, and we went through probably three or four DOH PIOs [Public Information Officers] over the course of a couple of years and I don’t know if they were being fired or they just threw up their hands and gave up. And the things we were told we couldn’t talk about; we would [tell] the media [to] call the state and I never heard back from the state. So, the communication was not good.”

Some participants from FDOH recalled instances in which they were compelled to withdraw communications or were prohibited from disseminating messages to avoid acting in opposition to the governor. In one instance, a county FDOH Director used community partners to spread messages they felt forbidden to communicate: “And we knew where the safe spaces were in terms of being able to communicate the information… just to make sure that we are still at the will of the governor.” To circumvent the issue of censorship, this participant recommended that independent groups without ties to the state government lead communication efforts.

Other concerns around the messenger included the lack of a clear authoritative source of information and the public’s invalidation of individuals communicating messages to the public. Participants across various sectors endorsed a messenger who was trusted and well-known in non-pandemic times. One public health academic suggested:

“Communication is key. In the book that they put together at the CDC they talk about communications and the need for a trusted leader that speaks to the science and not politics. They need to be trusted, and their position needs to be established early on.”

This individual or group should be able to positively convey information and sell a message to encourage public compliance during an emergency.

### Minimizing other adverse effects of a pandemic

#### Economic effects

Several participants, particularly those from government and the private sector, acknowledged the adverse economic effects associated with the COVID-19 pandemic and the corresponding response. For instance, a few participants from the hospitality industry linked closures and lockdowns to the loss of jobs and livelihoods. Essential workers, such as emergency room personnel, and lower-income individuals were said to be the most affected by public health interventions. These populations were often forced to decide between staying home to protect their health or working amid a pandemic to maintain financial security:

“Because at the end of the day, who went to work? The essential workers, the lowest paid people are the ones that continued. They had to get on the bus… and they had to take care of the basic needs of all of us.” (hospitality industry leader)

Interviewees from the private sector and government emphasized the importance of developing timely strategies to provide necessities to underserved populations in the event of lockdowns. One hospitality industry leader referred to the challenge of unemployment systems not being equipped to handle the volume of individual applications following mass layoffs and furloughs during COVID-19, which led to delayed processing of unemployment benefits. This participant advocated for streamlined processes, including employers providing registers of furloughed employees to unemployment offices.

Many small businesses were said to have endured the economic consequences of pandemic interventions due to financial losses associated with closures. To address these concerns, some decision-makers advised Florida to play a larger role in economic relief for small businesses through public assistance, which may include, for example, a commercial rent relief package. A few participants, including a state-level FDOH leader, a member of the Florida Legislature, and a hospitality industry leader, emphasized allowing businesses to remain open during a public health emergency to mitigate financial strains associated with closures, especially in consideration of Florida’s reliance on its service industry. One FDEM leader stressed, “…if you’re just dealing with Universal and Disney and Busch Gardens…they would probably have the resources to stay [shut] down for a month…A local restaurant [or] bar, a nightclub, doesn’t have those advantages.”

#### Other health priorities

While the pandemic response was intended to protect the population from the spread of COVID-19, participants warned that other health concerns inadvertently emerged as a result. Leaders from government and the hospitality industry pointed to the rise of mental health issues, such as depression and suicide, as well as the increased incidence of domestic violence following the implementation of closures and stay-at-home orders [54, 55]. One member of the Florida Legislature, for example, stressed “what we don’t want to do is substitute one public health crisis for another.” Participants from public health and the hospitality industry noted that the pandemic disrupted some people’s access to care for other health issues due to the prioritization of hospital capacity to address the virus and fears of contracting COVID-19 at these facilities:

“… I don’t know how they managed all these folks that have other illnesses, that needed to get treatments or prevent cancer spreading… they couldn’t even get access to the hospitals, there’s still other things going on, right?” (hospitality industry leader)

“People actually didn’t go to the hospital because they were afraid of catching it in the hospitals. I think some people died that should have been treated.” (county FDOH Director)

Participants across various sectors discussed how to balance the preservation of normalcy alongside pandemic mitigation strategies to promote the maintenance of mental and physical health as well as typical economic operations. A leader in hospitality argued for “[finding] a way to let people continue with their lives” by limiting or avoiding lockdowns and closures and allowing the public to assess their own individual risk. For instance, one participant from FDOH at the state-level proposed that school closures be restricted to short intervals as necessary, rather than long stretches of time.

### Public health culture

#### Prioritization of public health

Participants across all represented sectors highlighted the need to cultivate a culture that prioritizes public health and safety. One state senator found it difficult to convince leaders and the public to “get serious about pandemics” during COVID-19. A state-level FDOH leader recounted how other competing priorities, such as the economy, often engendered low prioritization of public health:

“The number one industry in Florida is tourism and recreation. So, if all the bar owners, businesses, resorts [are] saying ‘you’re killing us, we’re losing money,’ and these individuals are big donors to the administration, all of a sudden that puts a conflict in place [with public] health…”

Acknowledging the lack of political will to implement public health interventions, participants from FDOH and the state legislature noted the need to increase support for public health among decision-makers.

Among the public, participants from academia, public health, emergency management, and government noted that certain U.S. values, such as privacy and individual rights, sometimes took precedence over the need to protect the health of the collective, inhibiting the use of effective mitigation strategies. Prioritization of public health and the safety of the community was said to require the public’s acceptance of forgoing privacy in the event of a pandemic in order to allow for the use of more intrusive, yet effective surveillance strategies, such as those utilized in other countries:

“Well, the reason why we suffered with [testing, tracking, and isolating] in this country more than many other countries is because of our privacy laws and the truth of the matter is unless we get comfortable, in my opinion, with foregoing a tiny bit of privacy in the pandemic…” (FDEM leader)

Participants from academia, the state legislature, and the hospitality industry believed a change in culture is needed that prioritizes public health, and that greater public health literacy by means of long-term education campaigns is needed, highlighting the importance of preparing the public for future public health emergencies.

#### Trust

Trust in the science of public health and in the intentions of government officials was implicated in shaping the extent to which COVID-19 responses prioritized public health, and whether guidance was adhered to by the public. One academic said, “the trust in the public health system has been totally undermined. And I think that’s what worries me the most looking into the future.” Participants from all sectors interviewed suggested that scientific evidence on the severity of the public health crisis was at times accepted or rejected across partisan lines, explaining the State’s unwillingness to provide science-based recommendations:

“I think the second most difficult thing we faced was the politicization of the pandemic…all of a sudden there were two realities…that by far made it much more difficult for government to respond, for people to accept what government was doing, and for [public health officials] to mitigate the pandemic.” (FDEM leader)

Several participants expressed that the lack of trust in the guidance issued by public health officials and decision-makers—and in the idea that officials were acting in the public’s best interest—was tied to the public’s noncompliance with COVID-19 mitigation interventions. To combat these concerns and build trust, a few leaders from the state legislature and FDOH at the state and county levels recommended that individuals who are well-trusted by the public be tasked with validating scientific evidence. One state senator also pointed to the need to ensure data-driven decision-making and consistent collaboration with scientific experts.

## Discussion

This study is the first to empirically solicit multisectoral stakeholder insights on recommendations and challenges emerging from pandemic preparedness and response (PPR) in Florida. Analysis of participants’ input focused on recommendations for PPR, and challenges posed by pandemics across six subdomains—with examples of successes and failures during the COVID-19 pandemic in Florida. The framework presented represents an extensive range of interrelated topics of interest to stakeholders in Florida PPR, which may also be of broader interest to national and international public health leaders and policymakers. Reflecting on challenges from the COVID-19 pandemic, participants indicated the need to establish systems capacities and processes for PPR during non-emergency times. For an outbreak response to be successful, participants highlighted the importance of activating the appropriate actions in a timely manner, utilizing strategies built upon foundational public health capacities, established relationships among stakeholders, and effective communication across multiple levels. However, due to the diversity of participant responses, consensus was not found across recommendations. While there was some overlap, participants had different ideas on what the state of Florida should do to address PPR.

Success, therefore, was not defined only by the mitigation of disease transmission—for some it also included minimizing other adverse effects. While most participants focused solely on improving public health measures, others were concerned with the ramifications of policies on the economy and society. During the COVID-19 pandemic, economic and other health priorities were positioned as tradeoffs to public health, with differences in prioritization often stoking political polarization [56, 57]. Measures taken by governments to mitigate disease engendered consequences—around economy [58, 59], education [60, 61], and noncommunicable diseases [62]—contributing to a backlash and counter-narrative that further undermined public health objectives [63]. While the question of whether public health should eclipse economic concerns, or vice versa, has been widely contested and politicized [64–67], our study underscores the reality that pandemics pose an existential threat—to both life and livelihood. The collective views of participants in this study point to an imperative to address both health and economic priorities adequately and equitably during, and outside of pandemic times.

The multi-dimensionality of what constitutes a successful outbreak response undergirds the interplay of politics and public health in the United States during COVID-19. Participants called for consensus-building across “red and blue” and emphasized the importance of a unified response across political lines. Decisions that occurred in response to COVID-19 were not merely actions motivated by public health objectives, but also actions that were influenced by political affiliations [1, 6, 9, 20]. Our research aligns with the existing literature on the distinct approaches taken by Democratic and Republican governors during COVID-19, and the damaging consequences of such divergence [1, 6, 20–22, 68]. While Democratic party leaders were typically in favor of stronger pandemic mitigation efforts in place for longer periods, Republican leaders generally prioritized the preservation of economic activity and a quicker return to a sense of normalcy [1, 6].

As a Republican-led state dependent on its hospitality and tourism industry [16, 17], Florida often centered economic interests in decisions forgoing restrictions on businesses and individuals [5]. The issue of limited funding for public health, as highlighted in this study, is demonstrated by Florida’s ranking of 44 out of 49 states in dollars dedicated to public health per person [69] Limited growth in Florida’s public health funding between 2007 and 2019 highlights the reality of low prioritization that public health has historically engendered in the state [69]. Our findings align with other stakeholder-informed studies that highlight the importance of sustained funding for local health departments and pandemic preparedness [41, 43]. In Florida, low public health funding possibly reflects the population’s priorities and politics; one study, for example, found that 47.8 percent of the state’s registered Republican voters, who make up the majority of voters in the state [70], identified COVID-19 as a greater threat to the economy than to public health [71]. Acknowledging Florida’s unique economic needs, many participants advocated for a balanced and flexible pandemic response that does not position the economy and the protection of the collective at odds. Consensus-building is therefore necessary to address the objectives of all political persuasions while dispelling the narrative of public health and economy as zero sum tradeoffs.

Our results demonstrate the need for improved interjurisdictional collaboration, for which roles and authorities are pre-determined. As participants noted, there was tension between state and local authorities, which became tangible when the Florida governor issued an executive order suspending local COVID-19 mandates that impeded the “presumption of commercial operation and individual liberty” [11]. Participants from county health departments believed that the lack of a cooperative state-local approach obstructed their abilities to coordinate a public health response suitable for their localities. Similar studies and papers yielded varied responses on whether state pandemic responses should be centralized or decentralized [41, 42, 72]. While stakeholders from Delaware and Massachusetts mostly advocated for regionalized approaches and legal responses that include additional actors beyond the governor [41, 72], a Missouri-based study determined that their state’s decentralized approach prompted cross-jurisdictional confusion regarding who had decision-making authorities locally [42]. However, rather than recommending complete centralization of decision-making powers, stakeholders from Missouri stressed the need for clarification of their governance structure and local authorities as well as the creation of a mechanism to harmonize policies [42].

Further, several participants implied that the ability for local public health officials to be in a leadership position and communicate with the public was partially contingent on whether their recommendations were in line with the state’s approach to COVID-19, which was characterized by fewer restrictions. Some participants spoke to the need for greater autonomy and flexibility for local health authorities to implement crisis responses tailored to local community needs and contexts. They also discussed the challenge of a top-down approach for those at the local level. While states maintain the primary responsibility in responding to public health emergencies [1–3], a cooperative state-local approach explicitly establishing roles and responsibilities, including greater local input on decision-making ahead of future pandemics, would improve collaboration and help relieve tensions around authority.

Our study reveals the need for clarity in state agency roles pertaining to incident command, logistics, and provision of policy recommendations. Such clarity would help ease interagency tensions and ensure adequate preparedness for future pandemic response coordination. Participants spoke to confusion over roles and changes in incident command management that created friction between Florida Department of Health and Florida Division of Emergency Management and was exacerbated by the lack of infrastructure for interagency communication and coordination during the pandemic. While the current Florida Statutes designate the director of FDEM as the liaison to and coordinator of the Interagency Workgroup for “natural hazards,” there remains no provision for a unified command structure in the event of a public health emergency [73]. These gaps in coordination weakened the supply chain and contributed to the slow procurement of necessary public health goods in Florida. Interagency confusion around authority also created an unequal balance in participation between FDOH and FDEM. Although participants had varying perspectives on the responsibilities that each agency should assume, all pointed to the need for clearly defined roles and the development of infrastructure with clear leadership for public health emergencies to aid in communication and collaboration. Goldstein and Suder also stressed the need for improved coordination between the State of Delaware and relevant agencies, recommending the establishment of a core team or point person to manage inter-agency communication [72].

We joined other efforts to identify the lessons learned from the COVID-19 pandemic and to develop recommendations to better prepare for future public health emergencies [74–76]. The State of Florida took measures to improve preparedness for future public health emergencies through the instatement of a provision in the Florida Statutes that directed FDOH to collaborate with FDEM to develop a statewide public health emergency plan by July 2022 [77]. Based on the responses from stakeholders interviewed for this study, a pre-established plan or “playbook” should clearly outline the roles of each agency and the laws that must be waived to facilitate a stronger and more timely response. Accounting for Florida’s reliance on its service industry, the inclusion of a variety of stakeholder perspectives and economic considerations within the playbook can help balance the interests of emergency management, public health, and the economy, further engendering public trust and the separation of politics from public health.

### Limitations

This study provides a rich thematic analysis of stakeholder perspectives on PPR. However, there are several limitations. The study sample is limited to stakeholders from government, academia, and the private sector who were involved in the response to COVID-19 in Florida. While we aimed for representation from a larger proportion of localities in Florida, the sample represents 7 counties. Additionally, the sample was not intended to represent all stakeholders in Florida, nor those in other U.S. States, and therefore, is not generalizable in that way. The perspectives of lay Floridians, for example, were not directly solicited. Nonetheless, the participants offered insights on federal level PPR, and some of their state-level recommendations align with stakeholder-informed studies from other states [41–43]. We recognize the limitations of qualitative research [78–82], particularly in how the use of smaller samples limits the generalizability of the results to broader populations [82, 83]. However, rather than aiming for generalizable and universally applicable recommendations, this study seeks to generate valued insights from the specific participants interviewed, from which theory and additional research questions may be generated.

Due to challenges in initial recruitment of county-level Florida Department of Health Directors and Administrators, participant selection was broadened to include other stakeholders through snowball sampling. Considering the political climate in Florida and the incident involving one county FDOH Director being placed on leave for encouraging staff to get vaccinated [84], recruiting participants from local health departments to meet our initial goal proved to be a challenge. Consequently, non-response bias may be present. Additionally, as several participants currently worked in the state government, there may be limitations surrounding their candidness about state actions. However, given the diversity of responses provided in the interviews, as well as the overt critique of actions taken by decision-makers, response bias may be minimal. Future studies may provide further community-centric perspectives and discuss the experiences of other states.

### Conclusions and recommendations

The findings from this study emphasize the importance of soliciting stakeholder input to enhance policy making around state pandemic preparedness and response. Drawing from stakeholder perspectives on the challenges, failures, and successes encountered during Florida’s COVID-19 response, this study is the first to offer insights on what measures should be adopted ahead of the next pandemic to improve PPR in Florida. While our findings often aligned with those from similar stakeholder-informed studies, this study presents a broad range of recommendations, addressing both state and federal level considerations as well as implications for public health, the economy, and society. Beyond providing a detailed assessment of the state’s COVID-19 response, this study elicited several actionable recommendations that would allow Florida to better prepare for and respond to future public health emergencies. Participants revealed glaring deficiencies in the state’s public health systems capacities that must be addressed ahead of the next pandemic. Further, our study provides considerations around improving communications and balancing interests to create a public health response that is more palatable to a politically diverse public. The tensions between jurisdictions and agencies described by participants in this study and others, signifies the need for further research to gather more input from other states on the most effective approaches to designate authority and clarify roles.

Given the findings of this study, Florida policymakers are encouraged to heed the challenges and recommendations identified by stakeholders to improve the state’s response to future pandemics, potentially saving lives and livelihoods.

At the state level, decision-makers should consider the following recommendations:

- Funding- Increase state public health spending, including funding for county health departments.
- Workforce- Establish a statewide training curriculum to ensure consistency and a better prepared public health workforce.
- Planning- Enhance state pandemic plans through the identification of laws that should be waived at the onset of an outbreak to facilitate a timely response. This should include laws that limit the practice of out-of-state nurses and those that lengthen the process of procuring goods.
- Preparedness and Representation- Establish a Task Force that is aimed at addressing the gaps highlighted in our study and include input from stakeholders from public health and emergency management.
- Data Availability- Develop and maintain a dashboard with transparent and accurate data during a public health emergency to support public trust and informed decision-making.
- Interagency Cooperation- Clearly define the roles and responsibilities of FDOH and FDEM in a public health emergency. Establish an Interagency Workgroup and a coordinator for the group akin to that for “natural hazards” in the Public Health chapter of the Florida Statutes.
- Local Inclusion- Consider greater inclusion of local government in decision-making and allow county health departments to articulate views that oppose those of the state.

At the federal level, decision-makers should consider the following recommendations:

- State Spending- Immediately declare full reimbursement for state pandemic spending at the onset of an outbreak to enable states to respond more swiftly.
- Resources- Increase national production of public health goods and establish pre-negotiated platforms for state resource needs to address concerns around the competition for resources.
- Laboratory Capacities- Grant greater flexibility to academic institutions and state labs for the development of diagnostic tests to prevent gaps in testing and over-reliance on the CDC.
- Messaging- Recruit trusted communicators to act as public health messengers ahead of the next pandemic. Communicators should be prepared to speak to positive outcomes, validate science, and deliver messages absent of politics to help strengthen the public’s trust in science and public health.

## Supporting information

S1 File Interview guide. (PDF)
