# Supplementary material for: Preparing for the next pandemic: Reflections and recommendations from Florida
Source: PLoS One. 2024 Dec 2;19(12):e0314570. doi: 10.1371/journal.pone.0314570 (PMC11611121; doi:10.1371/journal.pone.0314570)
Supplement: S1 File — (PDF) [file pone.0314570.s001.pdf]

Semi-Structured Interview Guide
Florida Pandemic Preparedness Stakeholder Study

Introduction (Interviewer)

Thank you for taking the time to meet with us. Public health experts believe that pandemics will strike again in the not-too-distant future. We are working on this project to help Florida be better prepared to prevent human and economic losses from the next pandemic.

As University of Miami Miller School of Medicine researchers, we uphold the highest ethical standards to protect the information you share with us and its anonymity. With your permission, we would like to record our conversation ***to ensure we fully and correctly capture your expert views and opinions****.*

We will not link your name to any information you provide. You will have the option to review the reports that emerge from this study before they are released. ***Do you mind being recorded?***

Questions

1. At the beginning of the pandemic, faced with a novel virus, no one knew exactly what to do. *We would like to ask you today to* ***share your thoughts on how we can stay ahead of future pandemics****.*

2. Knowing what you know now, what do you think should be done the same for the next pandemic? What should be done differently?

3. What were the most significant challenges you faced in the response to COVID-19 in your role as ___?

4. As a Florida leader, what would you like to see for future pandemic preparedness and response? What are your thoughts are on how we can protect residents of Florida from future pandemics?

    a. In terms of the public health response to pandemics, what can be organized at the State level/ how can the State organize in the future?

    b. What would county public health department offices benefit from having at their disposal, provided by the State, in the event of another pandemic?

Interview Guidelines

I. Interviewing Tips

The interview is designed to collect data about the participant's views on the subject. The most important part is gathering complete, accurate data. To do this, allow the participant to share their ideas freely. Do not impose your own ideas on the issue. Seek to understand what the participant is trying to communicate, rather than make judgments about it such as agreeing or disagreeing. Do not prime the responder with leading questions that may shape the interviewee's responses.

Other tips include:

- *Keep questions open-ended (i.e., do not offer questions with a yes/no response)*
- *While the conversation can be flexible, consistency in the questions is important; use the interview guide to follow the predetermined questions*
- *Use the interviewee's word choice when possible*
- *Be careful to not impose your own ideas on the interviewee*
- *Ask the interviewee to clarify meaning, if necessary*
- *Time-permitting, ask all questions on the interview question guide*

Offer probes to gather more information, such as:

- *Can you say more about that?*
- *Would you explain further?*
- *Would you give me an example of what you mean?*
- *Tell me more about that/say more about that.*
- *Is there anything else?*

II. General Interviewing Tips

- *Be attentive to speakers*
- *Be purposeful about verbal responses to comments*
  - *Make short verbal responses such as "OK" and "Uh huh"*
  - *Avoid responses that imply judgment about the comments, such as "correct", "yes", or "I agree"*
- *Remain aware of your nonverbal communication*
- *Use value-neutral gestures*
  - *Use head nodding, lifting eyebrows, gesturing with hands, pointing, or leaning forward/away to connote encouragement but not judgment*
- *Use a 15-second pause to allow for thinking before responses to questions*
- *Rephrase or reiterate questions, as needed*
